# Bufalin Inhibits Melanoma Progression and Endothelial Angiogenic Phenotypes via AKT Signaling

**DOI:** 10.1002/cnr2.70674

**Published:** 2026-09-08

**Authors:** Xiao xuan Zhao, Ming xin Li, Jiang xue Qu

**Affiliations:** ^1^ Department of Dermatology Dalian Dermatosis Hospital Dalian Liaoning P. R. China

**Keywords:** bioinformatics, Bufalin, melanoma, p‐AKT

## Abstract

**Introduction:**

Bufalin (BF) exhibits anti‐tumor activity in many tumor types. However, there are few reports on BF in melanoma, and its pharmacological effects have not yet been elucidated. The present study aimed to investigate the effects of BF on melanoma and angiogenesis and to explore its possible mechanism.

**Methods:**

Cell viability in melanoma was assessed by Cell Counting Kit‐8 (CCK‐8) and colony formation assays. Flow cytometry was used to evaluate cell cycle and apoptosis. The migration and invasion were detected via the scratch test and transwell chamber migration assay. Bioinformatics analysis and molecular docking were performed to predict the potential BF target. Western blotting was conducted to discover the related protein expressions. To evaluate the antitumor activity of bufalin in vivo, subcutaneous xenograft models were established using A375 tumor cells.

**Results:**

BF had an inhibitory effect on the growth of melanoma cells and arrested cell cycles in the G2/M phase. Annexin V‐FITC/PI staining assay indicated that BF can promote apoptosis. BF also repressed human umbilical vein endothelial cell growth, migration, and tubular structure formation. Bioinformatics analysis suggested that BF inhibition in melanoma may be regulated by the PI3K‐AKT signaling pathway. Molecular docking results showed that BF forms a hydrogen bond with AKT1. Western blot analysis revealed that BF repressed the phosphorylated AKT protein level. Bufalin inhibits melanoma tumor growth in a xenograft mouse model.

**Conclusions:**

The study results demonstrated that BF exerts antitumor activity both in vitro and in vivo. Molecular docking and western blot analysis showed that BF plays an anti‐tumor role by downregulating the expression of p‐AKT.

## 1 Introduction

Malignant melanoma (MM) is a severe skin cancer derived from melanocytes. Its morbidity and mortality have been increasing [1]. Advanced melanoma has a very poor prognosis due to insensitivity to chemoradiotherapy, with a 5‐year survival rate of 32%–52% [2]. Alterations in the PI3K/AKT pathway occur in up to 70% of melanoma cases and are associated with disease progression. The PI3K/AKT pathway is necessary for several key endothelial cell (EC) functions, including cell growth, migration, survival, and vascular tone [3]. AKT1 promotes melanoma brain metastasis and angiogenesis, largely due to the phosphorylation and regulation of important downstream effectors that promote aspects of angiogenic signaling [4]. Bufalin (BF) is a cardiotonic steroid derived from toad venom (Chan Su) and a traditional Chinese medicine, which has been used for treating tumor recurrence and metastasis. It reportedly reduces the side effects of radiotherapy and chemotherapy and improves clinical efficacy and quality of life. BF has anti‐tumor effects on many types of solid tumors [5, 6].

To date, most studies on bufalin in melanoma have focused on apoptosis induction via caspase and mitochondrial pathways, or have identified individual binding targets [7, 8, 9]. None have systematically investigated whether bufalin acts through p‐AKT as a convergent node to coordinate both apoptosis and cell cycle arrest. Moreover, while AKT is frequently activated in both BRAF‐mutant and BRAF‐wild‐type melanomas [3], previous reports have not addressed whether bufalin's anti‐melanoma effects are mediated by p‐AKT independently of the BRAF background. Our study fills this gap by demonstrating, for the first time, that bufalin reduces p‐AKT protein levels in two BRAF‐mutant melanoma cell lines (A375 and A2058), and that this reduction is mechanistically linked to both increased apoptosis and G2/M phase arrest. Given that p‐AKT is a well‐established master regulator of cell death and cell cycle progression, our findings provide a new conceptual framework: bufalin acts as an upstream inhibitor of AKT phosphorylation, and this single event is sufficient to orchestrate downstream anti‐tumor programs.

## 2 Materials and Methods

### 2.1 Antibodies and Reagents

The primary antibodies and reagents used in the present study included PI3K (NO. 17366 s), AKT (NO. 4691), phosphorylated AKT (NO. 4060), and actin (NO. 3700). All antibodies were purchased from Cell Signaling Technology (Danvers, MA, USA). SC‐79 (Akt phosphorylation activator) was purchased from TargetMol. (Shanghai, China, T2274c), BF was obtained from Sigma‐Aldrich (Merck KGaA, Darmstadt, Germany). RIPA lysis buffer, enhanced chemiluminescence (ECL) kit, and bicinchoninic acid (BCA) protein assay were obtained from Beyotime Biotechnology Technology Co. Ltd., China. The phospho‐AKT antibody (CST #4060) detects AKT1 phosphorylated at Ser473 and also recognizes AKT2 and AKT3 phosphorylated at the corresponding residues; therefore, phosphorylated AKT is referred to as p‐AKT throughout this manuscript. BF was dissolved in DMSO, and the final concentration of DMSO in the culture medium was maintained at 0.1% (v/v). Control cells were treated with an equivalent concentration of DMSO.

### 2.2 Cells and Culture

Human melanoma cells A375 (BRAF gene mutation), A2058 (BRAF gene mutation), and HUVECs were obtained from the Chinese Academy of Sciences Committee Typical Culture Collection Cell Bank Shanghai, China. The cells were grown in RPMI‐1640 medium (Bioind) with 10% fetal bovine serum (FBS, Bioind) at 37°C with 5% CO_2_ in a humidified incubator. HUVECs were cultured in HUVEC‐specific medium (Procell Life Science & Technology Co. Ltd., Wuhan, China). HUVECs at passages 3–6 (P3–P6) were used for all experiments.

### 2.3 Cell Viability Assay

MM cells and HUVECs were seeded in 96‐well plates at a density of 5000 cells per well and incubated with various drug concentrations in triplicate for 24 h and 48 h. The cells were subjected to a Cell Counting Kit‐8 assay (Beyotime, Nanjing, China) according to the manufacturer's protocol. Then, the 450‐nm absorption value was used to determine the cell viability rate. The absorbance generated by WST‐8 formazan in untreated control cells was defined as 100% cell viability.

### 2.4 Annexin V‐FITC/PI Staining Assay

After treatment with various drug concentrations, cell apoptosis was detected using the annexin V‐FITC/PI apoptosis detection kit (Beyotime, Nanjing, China) following the manufacturer protocol and analyzed using FACScan flow cytometry (Becton, Dickinson and Company).

### 2.5 Cell Cycle Analysis

After treating cells with gradient concentrations of BF solution for 48 h, 0.5 mL of PI was added to the cells and incubated for 30 min in the dark. The samples were then analyzed by FACScan flow cytometry (BD Biosciences). Flowjo software (BD Biosciences, Bedford, MA, USA) was used to evaluate the cell cycle distribution.

### 2.6 Colony Formation Assay

The MM cells were plated in six‐well plates. After treatment with gradient concentrations of BF, cells were cultured in drug‐free medium for approximately 14 days. Medium was renewed twice weekly. The cells were fixed with cold paraformaldehyde and stained with crystal violet. The number of colonies was determined using ImageJ Software (https://imagej.net/Downloads), and representative images were obtained with Perfection V700 Scanner (Epson Corporation, Japan).

### 2.7 Transwell Assay

For the migration assay, the cells suspended in serum‐free medium were plated in the upper transwell (24 well) chamber and treated with BF. The lower side of the chamber was filled with conditioned medium containing 10% FBS. After 12 h incubation at 37°C, the cells that migrated to the bottom side of the membrane were fixed with cold methanol and stained with 0.1% crystal violet. For the invasion assay, the transwell membrane was coated with a mixture of Matrigel (BD Biosciences, Bedford, MA, USA; Matrigel: serum‐free medium = 1:8). The remaining procedures were the same as those utilized for the migration assay. The stained cells were counted using an Olympus light microscope (Olympus Corporation, Tokyo, Japan).

### 2.8 Wound Healing Assay

The cells were seeded in six‐well plates. After reaching confluency, a 1‐mm‐wide wound area was made by pipette tip on the surface of the wells. The cells were then incubated with serum‐free medium containing gradient concentrations of BF. After incubation for 12 h, cell migration was observed and images were obtained under an inverted microscope.

### 2.9 In Vitro Angiogenesis Assay

A 96‐well plate was coated with Matrigel and seeded with HUVECs. The HUVECs (4 × 10^4^ cells/well) were treated with gradient concentrations of BF for 24 h. The morphogenesis of tube networks was analyzed using an Olympus light microscope (Olympus Corporation). The tube formation numbers were also quantified by Image J software (https://imagej.net/Downloads).

### 2.10 Bioinformatics Analysis

Pharmapper service (http://lilab.ecust.edu.cn/pharmmapper/index.php) was used to predict possible BF target genes [10]. The melanoma genes were retrieved from the MalaCards (https://www.malacards.org/pages/info/) and GENECLIP (http://ci.smu.edu.cn/genclip3/analysis.php) databases. GENECLIP also served as the source for angiogenesis genes. BF‐related genes were obtained by utilizing data from all of the four datasets. Then, the genes were subjected to Gene Ontology (GO), Kyoto Encyclopedia of Genes and Genomes (KEGG) pathway, and protein–protein interaction (PPI) network analyses. GO analysis was performed using the WebGestalt website (http://www.webgestalt.org/option.php) [11]; KEGG pathways were constructed using DAVID (https://david.ncifcrf.gov/), and a PPI network was constructed using the STRING database (http://www.string‐db.org/) [12]. Data were visualized using R package ggplot2 and Cytoscape 3.7.2 software. Molecular docking was performed using Schrodinger software.

### 2.11 Western Blotting

The cells were grown in the 6‐well plate treated with gradient concentrations of BF solution for 48 h. MM cells were harvested, washed with phosphate‐buffered saline twice, and lysed using RIPA lysis buffer. The protein concentrations of the cell lysates were measured using the BCA protein assay kit. The protein samples (10–40 μg) were equally loaded on a sodium dodecyl sulfate‐polyacrylamide gel electrophoresis gel, electrophoresed, and transferred to polyvinylidene fluoride membranes. The membranes were incubated with different antibodies overnight at 4°C following 2 h of blocking with 5% non‐fat milk powder. On the second day, the membranes were incubated with secondary antibodies. The protein bands were visualized using the ECL kit. The gray intensity of the bands was quantified by Image J software (https://imagej.net/Downloads).

### 2.12 Immunohistochemistry

Immunohistochemical staining was performed using the streptavidin‐peroxidase method. Formalin‐fixed, paraffin‐embedded tumor tissues were sectioned at 4 μm, deparaffinized, rehydrated, and subjected to antigen retrieval in 10 mM Tris‐sodium citrate buffer (pH 6.0) at 95°C for 20 min. Endogenous peroxidase activity was blocked with 3% H_2_O_2_ for 10 min, followed by blocking with goat serum for 30 min. Sections were then incubated overnight at 4°C with primary antibodies against p‐AKT (1:500) and Ki‐67 (1:500). After washing, sections were incubated with a biotinylated secondary antibody‐HRP conjugate for 60 min at room temperature. Signals were visualized using 3,3′‐diaminobenzidine, and nuclei were counterstained with hematoxylin. Stained sections were examined and quantified under a light microscope.

### 2.13 Animal Xenograft Model and Bufalin Treatment

To evaluate the antitumor activity of bufalin in vivo, subcutaneous xenograft models were established using A375 tumor cells. Briefly, cells in the logarithmic growth phase were harvested, washed, and resuspended in sterile PBS or serum‐free medium. A total of 1 × 10^7^cells were injected subcutaneously into the right flank of 4–6 week old female BALB/c nude mice. When tumor volumes reached approximately 100 mm^3^, mice were randomly divided into the control and bufalin‐treated groups. Mice in the treatment group received bufalin at a dose of 1.5 mg/kg/day by intraperitoneal injection every day for 12 days. Whereas mice in the control group received an equal volume of vehicle. Tumor length and width were measured every 2 days using calipers, and tumor volume was calculated according to the formula: volume = length × width^2^/2. Body weight was monitored throughout the experiment to assess systemic toxicity. At the end of the treatment period, mice were euthanized, and tumors were excised, photographed, weighed, and subjected to further analysis.

### 2.14 Statistical Analysis

All experiments were performed in triplicate. Two selected groups were assessed by Student's *t*‐test. Multiple‐group comparisons were evaluated using one‐way analysis of variance. Tukey's post hoc test was used following analysis of variance. Data are expressed as the mean ± standard deviation. A *p*‐value of ≤ 0.05 was considered statistically significant. SPSS 19.0 and GraphPad Prism 7.0 were used to perform statistical analyses and to generate figures.

## 3 Results

### 3.1 BF Inhibits Melanoma Cell Growth and Promotes Apoptosis

**FIGURE 1:**
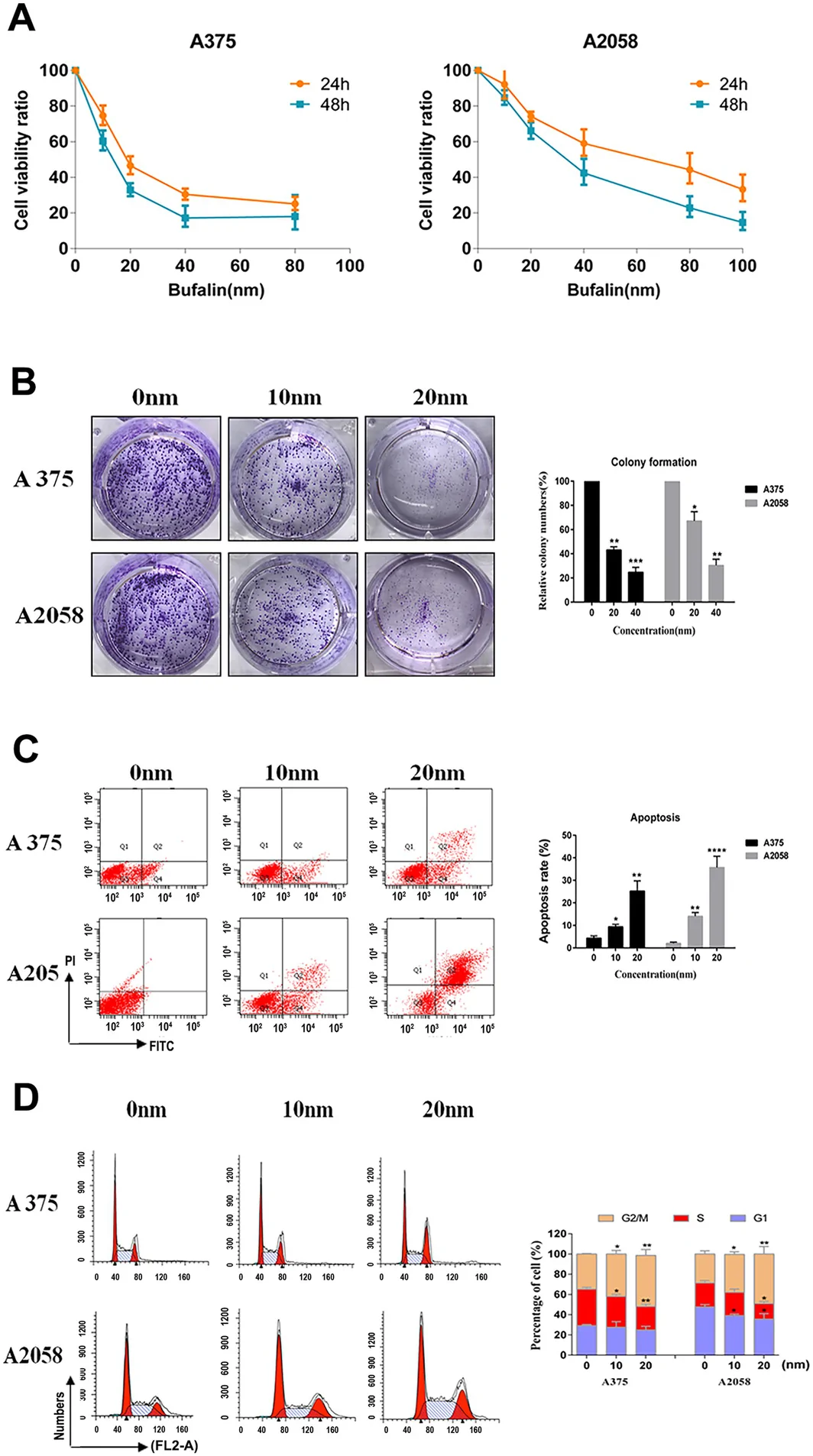
BF inhibits melanoma cell growth, promotes apoptosis, and arrests cell cycle in G2/M phase. (A) BF inhibits melanoma cell growth in a dose‐ and time‐dependent manner. (B) Number of formed colonies decreased significantly with increasing BF concentration. (C) After A375 and A2058 cells were treated with different concentrations of BF (0, 10, 20 nM) for 48 h, apoptotic cell numbers were significantly increased at concentrations of 10 and 20 nM. (D) Cell cycle results showing that the proportion of cells in G2/M phase increased with increased BF concentration, suggesting that BF arrests melanoma cells in G2/M phase.

BF inhibited the growth of melanoma cells in a dose‐ and time‐dependent manner. The 24‐ and 48‐h half‐maximal inhibitory concentration (IC50) values for BF in A375 cells were 20 nM (nmol/L) and 17.5 nM, respectively. The 24‐ and 48‐h IC50 values for BF in A2058 cells were 60 nM and 30 nM, respectively (Figure 1A). The colony formation of melanoma cells was significantly reduced with increasing BF concentration (Figure 1B). These results indicated that BF had an inhibitory effect on the growth of melanoma cells. Subsequently, the effect of BF on apoptosis and cell cycle of melanoma cells was examined using flow cytometry. The proportion of apoptotic cells was < 5% in the blank group and reached 10%–20% at 10 nM and 30%–40% at 20 nM in the treated group, indicating that BF can promote the apoptosis of melanoma cells (Figure 1C). The number of melanoma cells in the G2/M phase increased significantly when treated with 10‐nM and 20‐nM BF, suggesting that it can block melanoma cells in the G2/M phase (Figure 1D).

### 3.2 BF Inhibits Melanoma Cell Migration and Invasion

**FIGURE 2:**
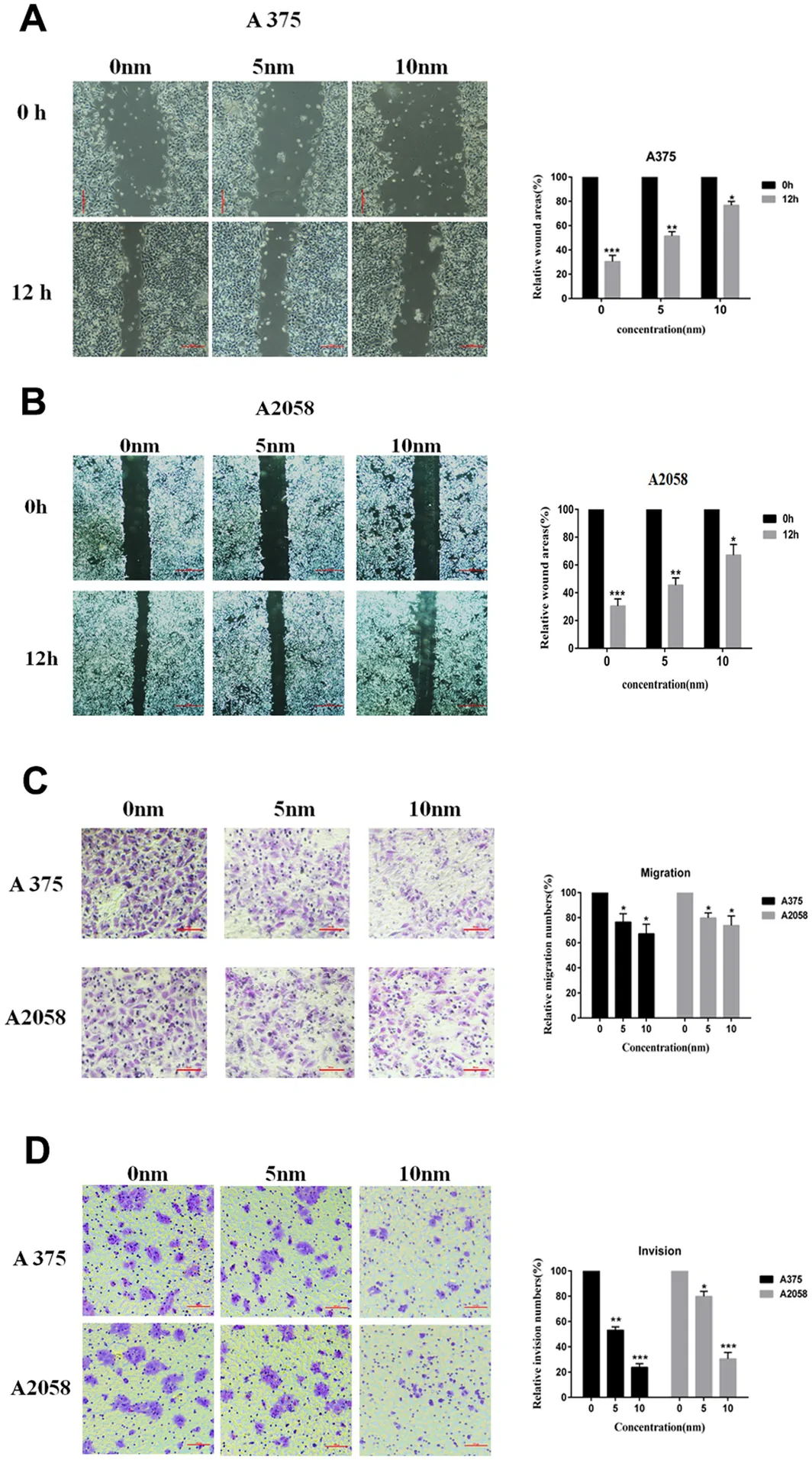
BF inhibits melanoma cell migration and invasion. Migration ability of melanoma cell lines was significantly weakened after treating A375 (A) and A2058 (B) cells with 5‐nM and 10‐nM BF for 12 h. (C) Number of melanoma cells migrating to the lower chamber was reduced after treatment with different concentrations 5, 10 nM of BF. (D) As the concentration of BF increased in transwell invasion assay, the invasive ability of melanoma cells was significantly weakened and the number of cells was significantly reduced.

The migration ability of melanoma cell lines A375 (Figure 2A) and A2058 (Figure 2B) was significantly weakened after BF treatment at a concentration of 10 nM. In addition, the scratches became wider. The effect of BF on the migration and invasion ability of melanoma cells was determined using a transwell assay. Compared to the control group, fewer cells transferred to the lower transwell chamber in the BF experimental group (Figure 2C). In the invasion experiment, the invasive ability of melanoma cells decreased and the number of cells was significantly reduced when the concentration of BF was 5 and 10 nM (Figure 2D).

### 3.3 BF Inhibits Growth, Migration, and Tubular Structure Formation of HUVECs

**FIGURE 3:**
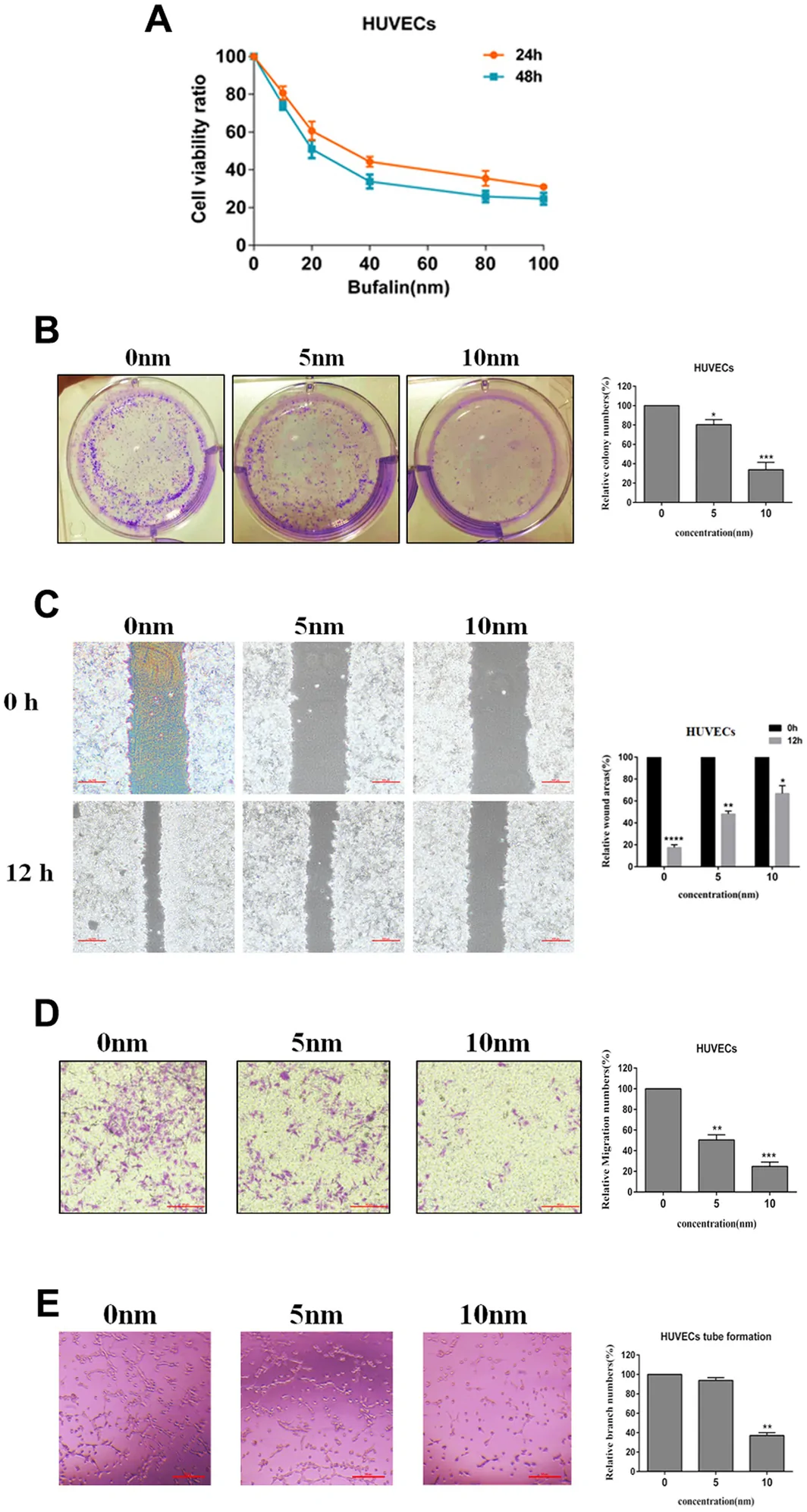
BF inhibits HUVEC growth, migration, and tubular structure formation. (A) HUVEC growth is inhibited with increasing BF concentration. (B) In the colony formation experiment, the number of colonies formed by HUVECs in drug‐treated group was reduced compared to control group. (C) Scratch spacing gradually widened after treating HUVECs with different concentrations 5, 10 nM of BF for 12 h. (D) Number of cells migrated to the lower chamber decreased significantly with increasing BF concentration. (E) After treating HUVECs with different BF concentrations (5, 10 nM) for 12 h, the number of formed dendritic tubules decreased, the structure was destroyed, and the number of formed tubular structures was significantly reduced.

In the CCK‐8 assay, BF inhibited the activity of HUVECs in a dose‐dependent manner with an IC50 of 40 nM at 24 h and an IC50 of 20 nM at 48 h (Figure 3A). Colony formation assays further confirmed the inhibitory effect of BF on the growth of HUVECs. Compared to the control group, the number of colonies formed by HUVECs was significantly reduced after adding BF (Figure 3B). In the wound healing assay, the wound width after adding BF was significantly greater in the experimental group than that in the blank control group at 5 and 10 nM (Figure 3C). Fewer cells migrated to the lower transwell chamber as the concentration of BF increased in the transwell assay (Figure 3D). Compared to the control group, the number of vascular networks formed by HUVECs at a concentration of 10 nM in the blood vessel formation experiment was significantly reduced, while the tubular structures were destroyed, indicating that BF inhibited the formation of HUVEC tubules (Figure 3E).

### 3.4 Bioinformatics Analysis Suggests That BF Inhibition in Melanoma May Be Regulated by the PI3K‐AKT Signaling Pathway

**FIGURE 4:**
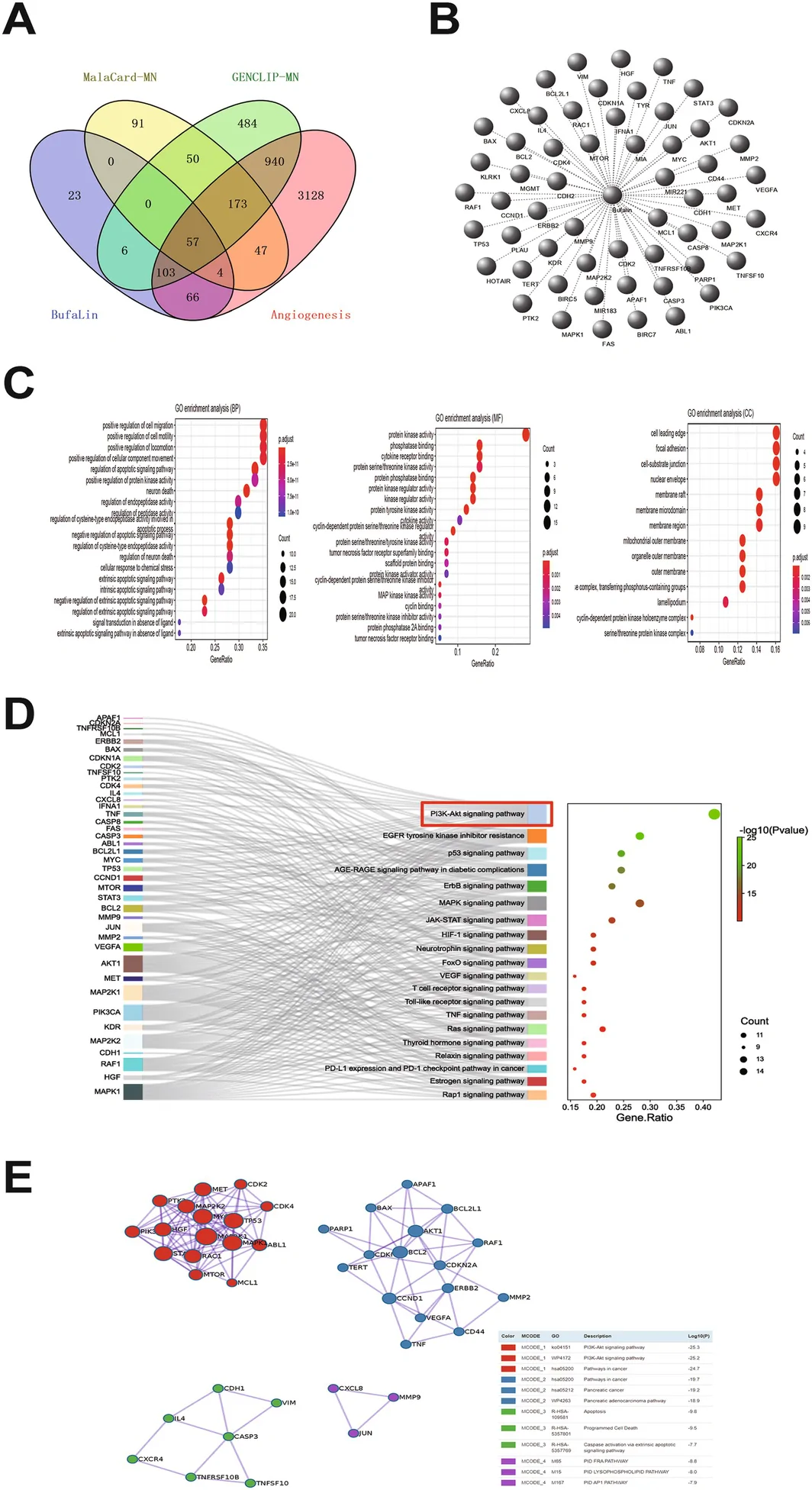
PI3K‐AKT signaling pathway is the main BF pathway for inhibiting melanoma growth. (A) A total of 57 common targets were obtained through Pharmmapper, MalaCards, and GENECLIP databases. (B) BF network and its target representation consisting of 57 nodes. BF is the middle molecule. (C) GO analysis results suggest that stimulation response and biological regulation were the main biological processes that affected protein regulation and cell and amino acid metabolism. Protein binding, ion binding, and transferase activity regulation are the main molecular functions of these genes. The genes are associated with various cellular components, including nucleus, cytoplasm, and cell membrane. (D) KEGG analysis screened 20 most important related pathways, and PI3K‐AKT pathway ranked first. (E) MCODE analysis suggested that PI3K‐AKT pathway was the most important signaling pathway.

The possible BF targets were obtained from the Pharmmapper database, while the melanoma and angiogenesis genes were retrieved from the MalaCards and GENECLIP databases [13]. Incorporating the data from the four databases, 57 key BF targets that may affect angiogenesis and melanoma growth were obtained (Figure 4A) and visualized using Cytoscape software (Figure 4B). GO analysis results showed that stimulation response and biological regulation were the main biological processes of BF‐related genes (Figure 4C). At the level of molecular function, protein binding, ion binding, and transferase activity regulation were the main molecular functions. These genes are associated with various cellular components, including the nucleus, cytoplasm, and cell membrane, mainly in protein‐bound form. KEGG analysis screened 20 related pathways, and the PI3K‐AKT pathway ranked first which regulated more key genes.

(Figure 4D). The MCODE modular analysis suggested that the PI3K‐AKT pathway was the most important signaling pathway (Figure 4E).

### 3.5 BF Reduces AKT Phosphorylation

**FIGURE 5:**
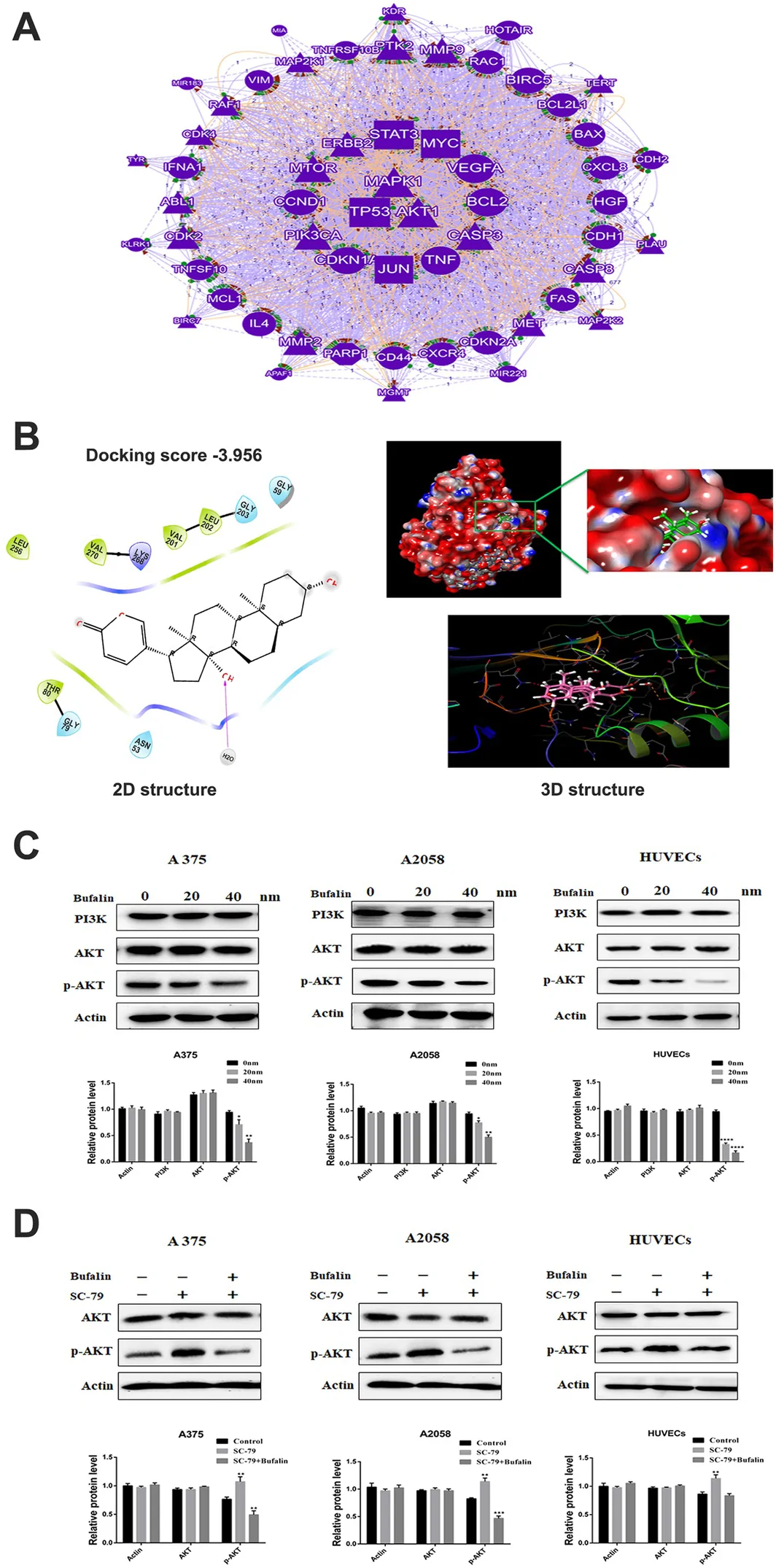
BF reduces AKT phosphorylation. (A) PPI protein interaction network analysis indicated that *MAPK*, *AKT1*, and *p53* were the core genes with the most interactions. (B) Molecular docking showed that BF and AKT1 formed a hydrogen bond at the 53rd asparagine site (ASN 53), and the docking score was −3.956. Results are represented in 2D and 3D. (C) Western blot experiments showed that protein levels of PI3K and total AKT did not change significantly, but p‐AKT protein levels decreased significantly. (D) Rescue experiment with SC‐79 showed that p‐AKT levels increased after SC‐79 was added. This increase was reversed by BF.

PPI protein interaction analysis was performed on related genes, among which *MAPK, AKT1*, and *p53* were the central genes that showed the most interaction (Figure 5A). Among these genes, AKT1 plays an important role in the PI3K‐AKT signaling pathway. Therefore, the study further focused of AKT1 molecules. Molecular docking simulated the molecular interaction between BF and AKT1. The molecular docking score represented the possibility of interaction between the two, such that the lower the score, the greater the possibility [14]. The score in the present study was −3.956, suggesting that BF interacts with AKT1, which was further verified using western blot experiments. There was no obvious change in the level of PI3K protein in melanoma cells and vascular endothelial cells after treatment with BF, but the level of p‐AKT protein was significantly reduced, indicating that BF reduces AKT phosphorylation (Figure 5C). Rescue experiments were then performed using SC‐79, which is an activator of AKT. The results showed that the expression level of p‐AKT protein increased after SC‐79 was added. This effect was reversed by BF (Figure 5D).

### 3.6 Bufalin Suppressed Tumor Growth in Xenograft Models

To determine whether bufalin inhibits tumor growth in vivo, A375 xenograft models were established in nude mice. Compared with the vehicle‐treated control group, bufalin treatment markedly suppressed tumor growth in both xenograft models (Figure S1A). Tumor volumes increased progressively in the control group, whereas tumors in the bufalin‐treated group grew more slowly and showed a significant reduction in final tumor size (Figure S1B). At the end of the experiment, tumors isolated from bufalin‐treated mice were visibly smaller than those from control mice. Consistently, tumor weights were significantly decreased following bufalin treatment (Figure S1C). No obvious loss of body weight was observed during the treatment period, suggesting that bufalin exerted antitumor effects without apparent systemic toxicity under the experimental conditions used. Immunohistochemical staining showed that bufalin treatment markedly reduced p‐AKT and Ki‐67 expression in tumor tissues compared with the control group (Figure S1D). Together, these results indicate that bufalin effectively inhibits the growth of A375 xenograft tumors in vivo.

## 4 Discussion

BF has demonstrated anti‐tumor activity in lung cancer [15], colorectal cancer [16], and breast cancer [17]. In signaling pathways that relate to the antitumor efficiency of Bufalin, PI3K/AKT, Hedgehog, MAPK/JNK, Wnt/β‐catenin, TGF‐β/Smad, Integrin signaling pathway, and NFKB signaling pathway were the most reported [18]. However, there have been few reports on BF in melanoma. The present study confirmed that BF had an inhibitory effect on MM cells and arrested the cell cycle in G2/M phase. In addition, BF induced apoptosis of melanoma cells. It has been previously reported that BF induces tumor cell apoptosis mainly via intracellular mitochondrial, extracellular death receptor, and endoplasmic reticulum mechanisms pathway [19]. The BF effect on apoptosis induction in tumor cells often occurs through multiple pathways and interactions that can be caspase‐dependent or ‐independent [20].

Tumor cell metastasis is an important reason for the high tumor lethality. Tumor cells can change their morphology, cross the basement membrane, and then migrate to other organs. Tumor cells will secrete cytokines and induce angiogenesis when they are transferred to a new environment [21, 22]. Neovascularization can increase the blood supply, provide nutrition, and maintain tumor growth. Therefore, inhibiting angiogenesis is one of the main methods to treat tumors. Wang et al. reported that BF can significantly inhibit the proliferation of vascular endothelial cells in liver cancer [23]. The present study found that BF inhibited the proliferation of vascular endothelial cells, induced apoptosis, and inhibited the formation of tubular structures in endothelial cells, indicating that it can inhibit angiogenesis in multiple ways.

In melanoma, the mechanism of anti‐tumor growth remains unclear. Soumoy et al. reported that BF inhibits the proliferation of melanoma cells via the Na +/K +/ATPase pump [24]. Pan et al. found that BF inhibits the proliferation, migration, and invasion of A375 melanoma cells by inhibiting the key enzyme PKM2 in the glycolysis pathway [25]. However, the above studies did not perform an in‐depth analysis of BF, and its pharmacological effects still need further research. In recent years, network pharmacology has provided a new guidance strategy for research. Network pharmacology is an interdisciplinary field integrating medicine, pharmacology, and bioinformatics. It is a discipline based on systems biology, which carries out network analysis on biological systems data and uses multi‐dimensional information for multi‐target drug molecule design [26]. In the present study, the online pharmacology and related biological information databases were used to screen out the target genes that BF acts upon in melanoma cells and vascular endothelial cells. Furthermore, bioinformatics analysis was used to enrich the PI3K‐AKT signaling pathway, which plays an important role in tumor growth and metastasis. The results suggested that BF inhibition in melanoma may be regulated by the PI3K‐AKT signaling pathway. In hepatocellular carcinoma, BF attenuates HIF‐1 by regulating the PI3KAKT pathway and reduces tumor metastasis [27]. BF inhibits the invasion and metastasis of lung cancer through the PI3K/AKT pathway [28]. It can also reverse the primary and acquired cisplatin resistance of gastric cancer by inhibiting the activation of the PI3‐K/AKT pathway [29]. Vascular endothelial growth factor (VEGF) is the most important regulator in the process of angiogenesis. It can enlarge the gap between cells in the vascular wall and facilitate its penetration by fibrinogen [30]. VEGF factor is regulated by the PI3K‐AKT signaling pathway [31], and BF can enhance the anti‐angiogenic effect of sorafenib by modulating the PI3K‐AKT‐VEGF signaling pathway [22].

PPI protein interaction analysis showed that BF may have an important effect on AKT protein. Molecular docking technology was used to simulate the binding of BF to AKT. Based on the characteristics of molecular structure, molecular docking provides an important technical means for drug design and prediction of functional sites by identifying the binding sites of interactions, which can simulate the interaction process between drugs and protein molecules [32]. The present results suggested that BF may affect the protein activity of AKT1 by forming a hydrogen bond with AKT1. It has also been reported that BF can reduce the resistance of sorafenib in liver cancer by inhibiting phosphorylated AKT and further enhance its anti‐tumor effect [33]. However, the prediction of drug target binding mode by molecular docking is only theoretical, and this theoretical model needs experimental support and verification. Western blot results in the present study showed that the level of phosphorylated AKT protein was significantly decreased after BF was added at different concentrations. Rescue experiments were performed with SC‐79, an activator of AKT which binds to its homologous domain and enhances its phosphorylation by upregulating protein kinases [34]. The results showed that p‐AKT levels increased after SC‐79 was added. This effect was reversed by BF. Combined with the molecular docking results, these outcomes demonstrate that BF may affect AKTprotein phosphorylation after forming a hydrogen bond with the 53rd asparagine site of AKT1 (ASN 53).

## 5 Conclusion

**FIGURE 6:**
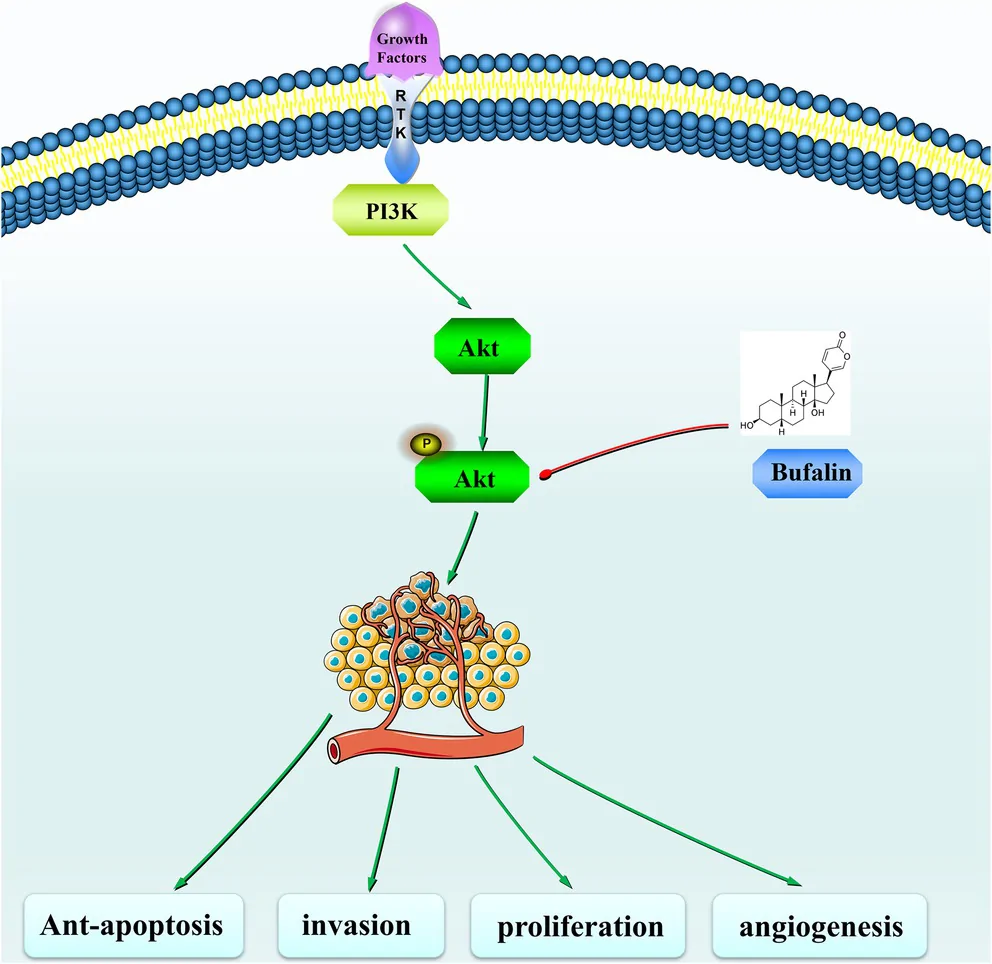
Schematic diagram of the antitumor effect of BF on melanoma. BF can inhibit the proliferation and invasion of melanoma cells, promote apoptosis, and hinder angiogenesis. The PI3K/AKT signaling pathway was downregulated by inhibiting AKT phosphorylation.

In summary, this study demonstrates that bufalin exerts anti‐melanoma effects by reducing p‐AKT protein levels, leading to both apoptosis and G2/M phase arrest in BRAF‐mutant melanoma cells (Figure 6). Given that AKT is a central kinase regulating cell survival and proliferation, our findings establish p‐AKT as a key mechanistic node for bufalin's action. The clinical applicability of these results is twofold. First, because p‐AKT downregulation is sufficient to trigger anti‐tumor effects regardless of BRAF mutation status, bufalin may be effective against a broad range of melanomas, including those resistant to BRAF/MEK inhibitors. Second, bufalin—as a natural compound already used in traditional Chinese medicine—could be repurposed as an adjunct therapy to enhance the efficacy of existing treatments or to overcome resistance. Future studies should explore the combination of bufalin with targeted agents or immunotherapies in preclinical models, as well as its safety and pharmacokinetics in melanoma patients. Overall, our work provides a mechanistic rationale for the clinical development of bufalin as a potential anti‐melanoma agent.

## Author Contributions

**Xiao xuan Zhao:** conceptualization, writing – original draft, writing – review and editing, funding acquisition, resources, supervision. **Ming xin Li:** software, methodology. **Jiang xue Qu:** software, methodology, formal analysis.

## Funding

This work was supported by the Project of the Western Medicine Bureau of Dalian Health Commission (No. 1911089).

## Ethics Statement

The authors have nothing to report.

## Consent

The authors have nothing to report.

## Conflicts of Interest

The authors declare no conflicts of interest.

## Supporting information


**Figure S1:** Bufalin inhibits melanoma tumor growth in a xenograft mouse model. (A) Representative photographs of dissected tumors from control (vehicle‐treated) and bufalin‐treated mice at the end of the experiment. (B) Tumor volume curves measured every other day over 27 days. Mice were treated with either vehicle (Control) or bufalin. Data are presented as mean ± SEM (*n* = number of mice per group). (C) Tumor weight at the endpoint. Each symbol represents an individual tumor. (D) Representative immunohistochemical staining of p‐AKT and Ki‐67 in tumor tissue sections from control and bufalin‐treated mice. Reduced p‐AKT and Ki‐67 signals are observed after bufalin treatment, indicating decreased AKT activation and cell proliferation. Scale bar = 50 μm. **p < 0.01, ***p < 0.001.

## Data Availability

The data that support the findings of this study are available on request from the corresponding author. The data are not publicly available due to privacy or ethical restrictions.

## References

[1] C. Pippa , M. Nicolas , B. Rossana , et al., “Comparative Efficacy and Safety of Targeted Therapies for BRAF‐Mutant Unresectable or Metastatic Melanoma: Results From a Systematic Literature Review and a Network Meta‐Analysis,” Cancer Treatment Reviews 110 (2022): 102463. 36099854 10.1016/j.ctrv.2022.102463

[2] D. Reichstein , A. Brock , C. Lietman , and M. McKean , “Treatment of Metastatic Uveal Melanoma in 2022: Improved Treatment Regimens and Improved Prognosis,” Current Opinion in Ophthalmology 33 (2022): 585–590. 36094043 10.1097/ICU.0000000000000905

[3] D. A. Kircher , K. A. Trombetti , M. R. Silvis , et al., “AKT1 Activates Focal Adhesion Kinase and Promotes Melanoma Brain Metastasis,” Molecular Cancer Research 17 (2019): 1787–1800. 31138602 10.1158/1541-7786.MCR-18-1372 PMC6726552

[4] M. Y. Lee , A. K. Luciano , E. Ackah , et al., “Endothelial AKT1 Mediates Angiogenesis by Phosphorylating Multiple Angiogenic Substrates,” Proceedings of the National Academy of Sciences of the United States of America 111 (2014): 12865–12870. 25136137 10.1073/pnas.1408472111 PMC4156707

[5] H. Y. Jiang , H. M. Zheng , C. Xia , et al., “The Research Progress of Bufalin in the Treatment of Hepatocellular Carcinoma,” Oncotargets and Therapy 15 (2022): 291–298. 35345394 10.2147/OTT.S333233 PMC8957335

[6] L. Soumoy , G. E. Ghanem , S. Saussez , and F. Journe , “Bufalin for an Innovative Therapeutic Approach Against Cancer,” Pharmacological Research 184 (2022): 106442. 36096424 10.1016/j.phrs.2022.106442

[7] L. Soumoy , A. Genbauffe , L. Mouchart , et al., “ATP1A1 Is a Promising New Target for Melanoma Treatment and Can Be Inhibited by Its Physiological Ligand Bufalin to Restore Targeted Therapy Efficacy,” Cancer Cell International 24 (2024): 8. 38178183 10.1186/s12935-023-03196-y PMC10765859

[8] I. Pascual Alonso , L. Rivera Méndez , F. Almeida García , et al., “Bufadienolides Preferentially Inhibit Aminopeptidase N Among Mammalian Metallo‐Aminopeptidases; Relationship With Effects on Human Melanoma MeWo Cells,” International Journal of Biological Macromolecules 28 (2023): 825–837. 10.1016/j.ijbiomac.2022.12.280 36592847

[9] J. Liu , J. Jiang , J. Huang , et al., “Bufalin Inhibits Hepatocellular Carcinoma Progression by Blocking EGFR‐Mediated RAS‐RAF‐MEK‐ERK Pathway Activation,” Journal of Experimental & Clinical Cancer Research 44 (2025): 260. 40883741 10.1186/s13046-025-03531-3 PMC12395921

[10] X. Zhao , J. Liu , L. Yang , et al., “Beneficial Effects of Mijianchangpu Decoction on Ischemic Stroke Through Components Accessing to the Brain Based on Network Pharmacology,” Journal of Ethnopharmacology 285 (2022): 114882. 34848358 10.1016/j.jep.2021.114882

[11] Y. Liao , J. Wang , E. J. Jaehnig , Z. Shi , and B. Zhang , “WebGestalt 2019: Gene Set Analysis Toolkit With Revamped UIs and APIs,” Nucleic Acids Research 47 (2019): W199–W205. 31114916 10.1093/nar/gkz401 PMC6602449

[12] D. Szklarczyk , J. H. Morris , H. Cook , et al., “The STRING Database in 2017: Quality‐Controlled Protein‐Protein Association Networks, Made Broadly Accessible,” Nucleic Acids Research 45 (2017): D362–D368. 27924014 10.1093/nar/gkw937 PMC5210637

[13] Z. Wu , L. He , L. Wang , and L. Peng , “Systematically Exploring the Antitumor Mechanisms of Core Chinese Herbs on Hepatocellular Carcinoma: A Computational Study,” Evidence‐based Complementary and Alternative Medicine: eCAM 2020 (2020): 2396569.33014099

[14] Q. Zhang , L. Chen , M. Gao , S. Wang , L. Meng , and L. Guo , “Molecular Docking and in Vitro Experiments Verified That Kaempferol Induced Apoptosis and Inhibited Human HepG2 Cell Proliferation by Targeting BAX, CDK1, and JUN,” Molecular and Cellular Biochemistry 478 (2023): 767–780. 36083512 10.1007/s11010-022-04546-6

[15] J. Jin , Z. Yao , H. Qin , K. Wang , and X. Xin , “Bufalin Inhibits the Malignant Development of Non‐Small Cell Lung Cancer by Mediating the circ_0046264/miR‐522‐3p Axis,” Biotechnology Letters 43 (2021): 1229–1240. 33534015 10.1007/s10529-021-03081-6

[16] Z. Yuan , C. Liu , Y. Sun , et al., “Bufalin Exacerbates Photodynamic Therapy of Colorectal Cancer by Targeting SRC‐3/HIF‐1alpha Pathway,” International Journal of Pharmaceutics 624 (2022): 122018. 35839982 10.1016/j.ijpharm.2022.122018

[17] F. Chen , L. Zhu , J. Hu , et al., “Bufalin Attenuates Triple‐Negative Breast Cancer Cell Stemness by Inhibiting the Expression of SOX2/OCT4,” Oncology Letters 20 (2020): 171. 32934738 10.3892/ol.2020.12028 PMC7471667

[18] X. Zhang , X. Zhao , K. Liu , et al., “Bufalin: A Systematic Review of Research Hotspots and Antitumor Mechanisms by Text Mining and Bioinformatics,” American Journal of Chinese Medicine 48 (2020): 1633–1650. 33148004 10.1142/S0192415X20500810

[19] J. Song , D. Zou , X. Zhao , et al., “Bufalin Inhibits Human Diffuse Large B‐Cell Lymphoma Tumorigenesis by Inducing Cell Death Through the Ca2+/NFATC1/cMYC Pathway,” Carcinogenesis 42 (2021): 303–314. 33124657 10.1093/carcin/bgaa108

[20] H. R. LingHu , H. Luo , and L. Gang , “Bufalin Induces Glioma Cell Death by Apoptosis or Necroptosis,” Oncotargets and Therapy 13 (2020): 4767–4778. 32581545 10.2147/OTT.S242567 PMC7274536

[21] K. Fang , Y. Zhan , R. Zhu , et al., “Bufalin Suppresses Tumour Microenvironment‐Mediated Angiogenesis by Inhibiting the STAT3 Signalling Pathway,” Journal of Translational Medicine 19 (2021): 383. 34496870 10.1186/s12967-021-03058-z PMC8424978

[22] H. Wang , C. Zhang , H. Chi , and Z. Meng , “Synergistic Anti‐Hepatoma Effect of Bufalin Combined With Sorafenib via Mediating the Tumor Vascular Microenvironment by Targeting mTOR/VEGF Signaling,” International Journal of Oncology 52 (2018): 2051–2060. 29620259 10.3892/ijo.2018.4351

[23] H. Wang , C. Zhang , Z. Ning , L. Xu , X. Zhu , and Z. Meng , “Bufalin Enhances Anti‐Angiogenic Effect of Sorafenib via AKT/VEGF Signaling,” International Journal of Oncology 48 (2016): 1229–1241. 26782953 10.3892/ijo.2016.3326

[24] L. Soumoy , M. Wells , A. Najem , et al., “Toad Venom Antiproliferative Activities on Metastatic Melanoma: Bio‐Guided Fractionation and Screening of the Compounds of Two Different Venoms,” Biology 9 (2020): 218. 32785105 10.3390/biology9080218 PMC7464305

[25] Z. Pan , X. Zhang , P. Yu , et al., “Cinobufagin Induces Cell Cycle Arrest at the G2/M Phase and Promotes Apoptosis in Malignant Melanoma Cells,” Frontiers in Oncology 9 (2019): 853. 31552178 10.3389/fonc.2019.00853 PMC6738445

[26] T. T. Luo , Y. Lu , S. K. Yan , X. Xiao , X. L. Rong , and J. Guo , “Network Pharmacology in Research of Chinese Medicine Formula: Methodology, Application and Prospective,” Chinese Journal of Integrative Medicine 26 (2020): 72–80. 30941682 10.1007/s11655-019-3064-0

[27] P. Xia and X. Y. Xu , “PI3K/AKT/mTOR Signaling Pathway in Cancer Stem Cells: From Basic Research to Clinical Application,” American Journal of Cancer Research 5 (2015): 1602–1609. 26175931 PMC4497429

[28] Z. Zhu , H. Sun , G. Ma , et al., “Bufalin Induces Lung Cancer Cell Apoptosis via the Inhibition of PI3K/AKT Pathway,” International Journal of Molecular Sciences 13 (2012): 2025–2035. 22408435 10.3390/ijms13022025 PMC3292004

[29] H. Zhao , D. Zhao , H. Jin , et al., “Bufalin Reverses Intrinsic and Acquired Drug Resistance to Cisplatin Through the AKT Signaling Pathway in Gastric Cancer Cells,” Molecular Medicine Reports 14 (2016): 1817–1822. 27357249 10.3892/mmr.2016.5426

[30] S. H. Choi , S. S. Yoo , S. Y. Lee , and J. Y. Park , “Anti‐Angiogenesis Revisited: Reshaping the Treatment Landscape of Advanced Non‐Small Cell Lung Cancer,” Archives of Pharmacal Research 45 (2022): 263–279. 35449345 10.1007/s12272-022-01382-6

[31] J. M. Lu , Z. Z. Zhang , X. Ma , S. F. Fang , and X. H. Qin , “Repression of microRNA‐21 Inhibits Retinal Vascular Endothelial Cell Growth and Angiogenesis via PTEN Dependent‐PI3K/AKT/VEGF Signaling Pathway in Diabetic Retinopathy,” Experimental Eye Research 190 (2020): 107886. 31759996 10.1016/j.exer.2019.107886

[32] S. Ahmed , A. E. Prabahar , and A. K. Saxena , “Molecular Docking‐Based Interactions in QSAR Studies on *Mycobacterium tuberculosis* ATP Synthase Inhibitors,” SAR and QSAR in Environmental Research 33 (2022): 289–305. 35532308 10.1080/1062936X.2022.2066175

[33] H. Wang , C. Zhang , L. Xu , et al., “Bufalin Suppresses Hepatocellular Carcinoma Invasion and Metastasis by Targeting HIF‐1alpha via the PI3K/AKT/mTOR Pathway,” Oncotarget 7 (2016): 20193–20208. 26958938 10.18632/oncotarget.7935 PMC4991447

[34] D. Chang , J. Feng , H. Liu , W. Liu , L. Sharma , and C. S. Dela Cruz , “Differential Effects of the AKT Pathway on the Internalization of Klebsiella by Lung Epithelium and Macrophages,” Innate Immunity 26 (2020): 618–626. 32762278 10.1177/1753425920942582 PMC7556185

